# Immunological imprinting of humoral immunity to SARS-CoV-2 in children

**DOI:** 10.1038/s41467-023-39575-2

**Published:** 2023-06-29

**Authors:** Alexander C. Dowell, Tara Lancaster, Rachel Bruton, Georgina Ireland, Christopher Bentley, Panagiota Sylla, Jianmin Zuo, Sam Scott, Azar Jadir, Jusnara Begum, Thomas Roberts, Christine Stephens, Shabana Ditta, Rebecca Shepherdson, Annabel A. Powell, Andrew J. Brent, Bernadette Brent, Frances Baawuah, Ifeanyichukwu Okike, Joanne Beckmann, Shazaad Ahmad, Felicity Aiano, Joanna Garstang, Mary E. Ramsay, Rafaq Azad, Dagmar Waiblinger, Brian Willett, John Wright, Shamez N. Ladhani, Paul Moss

**Affiliations:** 1 Institute of Immunology & Immunotherapy, College of Medical and Dental Sciences, University of Birmingham, Birmingham, UK; 2 Immunisation Department, UK Health Security Agency, 61 Colindale Avenue, London, UK; 3 MRC-University of Glasgow Centre for Virus Research, Glasgow, UK; 4 Bradford Institute for Health Research, Bradford Teaching Hospitals NHS Foundation Trust, Bradford, UK; 5 Oxford University Hospitals NHS Foundation Trust, Old Road, Oxford, UK; 6 University of Oxford, Wellington Square, Oxford, OX1 2JD UK; 7 University Hospitals of Derby and Burton NHS Foundation Trust, Uttoxeter New Road, Derby, UK; 8 East London NHS Foundation Trust, 9 Allie Street, London, UK; 9 Manchester University NHS Foundation Trust, Oxford Road, Manchester, UK; 10 Birmingham Community Healthcare NHS Trust, Holt Street, Aston, UK

**Keywords:** Antibodies, Infection, Viral infection, Immunological memory, T cells

## Abstract

Omicron variants of SARS-CoV-2 are globally dominant and infection rates are very high in children. We measure immune responses following Omicron BA.1/2 infection in children aged 6-14 years and relate this to prior and subsequent SARS-CoV-2 infection or vaccination. Primary Omicron infection elicits a weak antibody response with poor functional neutralizing antibodies. Subsequent Omicron reinfection or COVID-19 vaccination elicits increased antibody titres with broad neutralisation of Omicron subvariants. Prior pre-Omicron SARS-CoV-2 virus infection or vaccination primes for robust antibody responses following Omicron infection but these remain primarily focussed against ancestral variants. Primary Omicron infection thus elicits a weak antibody response in children which is boosted after reinfection or vaccination. Cellular responses are robust and broadly equivalent in all groups, providing protection against severe disease irrespective of SARS-CoV-2 variant. Immunological imprinting is likely to act as an important determinant of long-term humoral immunity, the future clinical importance of which is unknown.

## Abstract (web summary)

The immune responses to SARS CoV-2 infection in children are less well understood than in adults. Here the authors characterise immune responses to newer omicron lineages and relate these to previous infection with earlier lineages of SARS-CoV-2, implicating a reduced immunogenicity from omicron variants and imprinting from previous virus strains.

## Introduction

The emergence of the SARS-CoV-2 Omicron variant (B.1.1.529) marked a major shift in the COVID-19 pandemic. The acquisition of multiple mutations, primarily within the spike receptor binding domain (RBD), enhanced Omicron infectivity and led to substantial evasion of antibody responses elicited from prior infection or vaccination^1–3^. As such, the variant demonstrated a remarkable capacity to outcompete pre-Omicron SARS-CoV-2 and became the dominant variant globally.

Omicron infection rates have been high in all age groups irrespective of prior infection or vaccination status but have been particularly notable in children^4,5^. Reinfection with Omicron subvariants is also observed^6^ and protection against reinfection appears lower than was seen in children following infection with pre-Omicron SARS-CoV-2^7^. Studies of children who have been infected with pre-Omicron SARS-CoV-2 virus or have undergone vaccination reveal relatively low antibody binding to Omicron, in a similar pattern to that seen in adults^1,8–10^. Less is known regarding the nature of the immune response elicited by primary Omicron infection in this age group, although low antibody responses have been reported^11^ and concur with similar findings in adults^12,13^. Prior infection status with pre-Omicron variants is also suggested to be an important determinant of response to Omicron in adults^8,13,14^.

Profound differences are observed in the age-dependency of the immune response against SARS-CoV-2. Infections are generally mild or asymptomatic in children and potential determinants of this may include increased expression of IFN-response genes within the mucosal epithelium and enhanced innate immune responses^15,16^. Reassuringly, children develop robust systemic adaptive immune responses against pre-Omicron variants, even after asymptomatic infection^17^, with increased clonality compared to adults^16^, although it is not known if similar immune responses will be observed after Omicron infection.

Here we study antibody and cellular immunity in children following a recent Omicron infection. Furthermore, we relate immune response to prior infection or vaccination and also assess the subsequent impact of reinfection or vaccination. Primary Omicron infection is weakly immunogenic whilst the profile of humoral response is markedly influenced by prior infection or vaccination and matures substantially following reinfection. Cellular immunity is robust in all cases. These data have important implications for understanding the immune responses against SARS-CoV-2 in children and guiding optimal immune protection.

## Results

### Primary Omicron infection in children elicits low antibody titre with poor neutralisation activity

Blood samples from the sKIDS (*n* = 29) and Born in Bradford (*n* = 14) cohort studies were available from 43 unvaccinated children who had recently tested positive for Omicron BA.1/2 infection (Supplementary Fig. 1 and Supplementary Table 1). The sKIDs study of children aged 6–14 years^18^ and the Born in Bradford (BiB) study of children aged 8–15 years are blood sampling studies of children during the COVID-19 pandemic and their design and sampling dates are explained fully in the Methods section^19^.

**Fig. 1:**
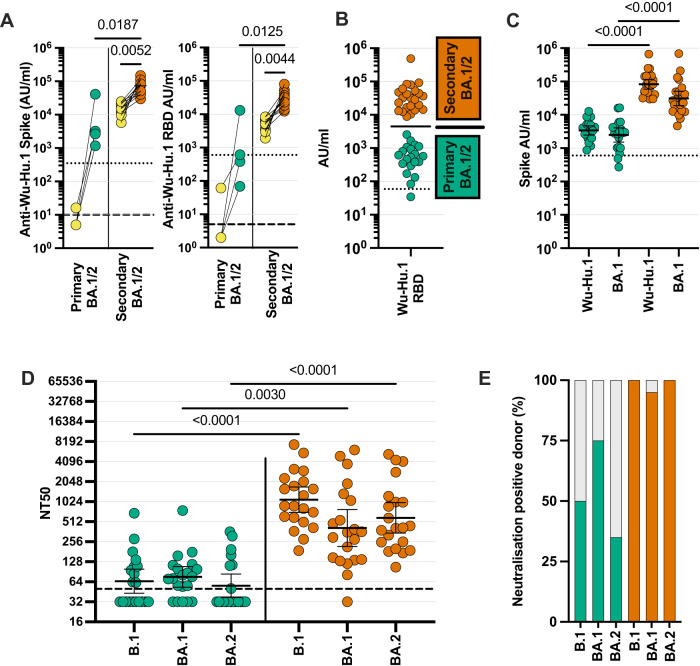
Low titre antibodies and neutralisation develop following primary Omicron infection. SARS-CoV-2-specific antibody responses were determined in children with recent Omicron BA.1/2 infection. **A** Wuhan-Hu-1 (Wu-Hu-1) spike antibody binding in samples from children who were SARS-CoV-2 seronegative (*n* = 4) or seropositive (*n* = 11) prior to Omicron infection (yellow). Dotted lines indicate seropositive cut-off for Wuhan-Hu-1-specific antibody response; dashed lines indicate below the limit of detection. Data were presented as arbitrary units (AU)/ml. One-way Friedman Test with Dunn’s multiple comparisons test. **B** Wuhan-Hu-1 RBD-specific antibody binding in the total cohort of 43 children with recent Omicron BA.1/2 infection shows a bimodal distribution of primary (green; *n* = 20) or secondary (orange; *n* = 23) SARS-CoV-2 infection status. Data were presented as arbitrary units (AU)/ml. Bar indicates geometric mean; representative of primary or secondary Omicron infection. **C** Wuhan-Hu-1 and Omicron BA.1 spike-specific antibody binding in children with primary (green; *n* = 20) or secondary (orange; *n* = 23) Omicron BA.1/2 infection. The dotted line indicates seropositive cut-offs for Wuhan-Hu-1. Data were presented as arbitrary units (AU)/ml. One-way Kruskal–Wallis test with Dunn’s multiple comparisons test, bars indicate geometric mean ±95% CI. **D** Neutralisation of pseudovirus-bearing B.1 (Wuhan-Hu-1 and D614G) ancestral or Omicron BA.1 or BA.2 sequence spike protein in children following primary (green; *n* = 20) or secondary (orange; *n* = 21) Omicron infection. Dashed lines indicate the limit of detection. Data were presented as NT50, the titre at which infectivity had been reduced by 50% relative to controls. One-way Kruskal–Wallis test with Dunn’s multiple comparisons test, bars indicate geometric mean ±95% CI. **E** Bar graph indicating the percentage of children with detectable neutralising titres to B.1 or Omicron BA.1 or BA.2 following primary (green, *n* = 20) or secondary (orange, *n* = 21) Omicron infection. Source data are provided as a Source Data file.

Initial studies were undertaken to assess how antibody responses following Omicron compared in children in whom this represented their primary SARS-CoV-2 infection or those who had previously had a pre-Omicron infection. Serological samples prior to the Omicron infection were available from 15 children of whom four were SARS-CoV-2 seronegative (primary infection) and 11 seropositive (secondary infection). Primary Omicron infection elicited low antibody titres against both the ancestral spike and RBD domains (Fig. 1A and Supplementary Fig. 2A), consistent with previous reports^11^. Titres against the Delta variant were high in one child, likely due to subclinical Delta infection between blood sampling, and as such this was regarded as a secondary infection (Supplementary Fig. 2A). In contrast to the profile of primary infection, all secondary Omicron infections elicited a substantially higher antibody response.

This bimodal profile of antibody response was also apparent across the total cohort of 43 children with recent Omicron infection and was therefore used to define primary (*n* = 20) and secondary (*n* = 23) infection (Fig. 1B and Supplementary Fig. 2B). Due to the cross-sectional nature of the collection, samples were taken at different timepoints following infection but this did not impact on antibody titre (Supplementary Fig. 3A).

The magnitude and relative antibody response to both the ancestral or Omicron BA.1 infection was then determined following primary or secondary infection (Fig. 1C and Supplementary Fig. 4). Primary Omicron infection elicited broadly equivalent antibody responses against all variants although these were of low titre. Notably, Omicron-specific titres did not exceed those seen in historical samples following infection with a pre-Omicron variant (Supplementary Fig. 4). In contrast, antibody responses after secondary Omicron infection were 24-fold and 12-fold higher against ancestral and Omicron BA.1 spike (Supplementary Table 2).

Functional humoral immunity was assessed with a pseudovirus-based neutralisation assay following primary (*n* = 20) or secondary (*n* = 21) Omicron infection. Neutralisation capacity was low following primary infection and it was notable that 25 and 65% of children did not have a measurable neutralisation titre against BA.1 or BA.2 respectively. In contrast, neutralisation was robust following secondary infection (Fig. 1D, E).

These data show that primary Omicron infection in children elicits a low titre antibody response with poor neutralising activity. In contrast, prior infection with a pre-Omicron virus primes the immune system to develop robust humoral immunity to all viral variants following Omicron infection.

### Reinfection or COVID-19 vaccination markedly boosts antibody responses following primary Omicron infection

The endemic human coronaviruses OC43, HKU-1, 229E and NL63 (HCoV) are common aetiological agents of upper respiratory tract infection in children and repeated infection acts to incrementally elevate virus-specific antibody titres^20^. As such, we next assessed whether an Omicron reinfection could similarly act to boost humoral immunity following primary Omicron infection. The serological response to COVID-19 vaccination following primary infection was also determined.

Prospective follow-up serum samples were obtained from 24 unvaccinated children with prior serological evidence of primary Omicron infection. Omicron reinfection was identified on the basis of a >2-fold increase in spike-specific antibody titre, similar to previous reports of HCoV epidemiology^21^. Reinfection was observed in 13 children (54%), consistent with reported reinfection rates following primary Omicron infection^6^. Spike-specific antibody titres increased markedly following Omicron reinfection (>0.05, Kruskal–Wallis test, with Dunn’s correction) with titres against BA.1 increasing tenfold (Supplementary Table 2).

Samples were also obtained from 11 children who had received a single dose of the BNT162b2 vaccine following primary Omicron infection. A strong boosting effect was seen with BA.1-specific titres increasing by 115-fold following vaccination (>0.0001, Kruskal–Wallis test, with Dunn’s correction; Supplementary Table 2 and Supplementary Fig. 4C).

**Fig. 2:**
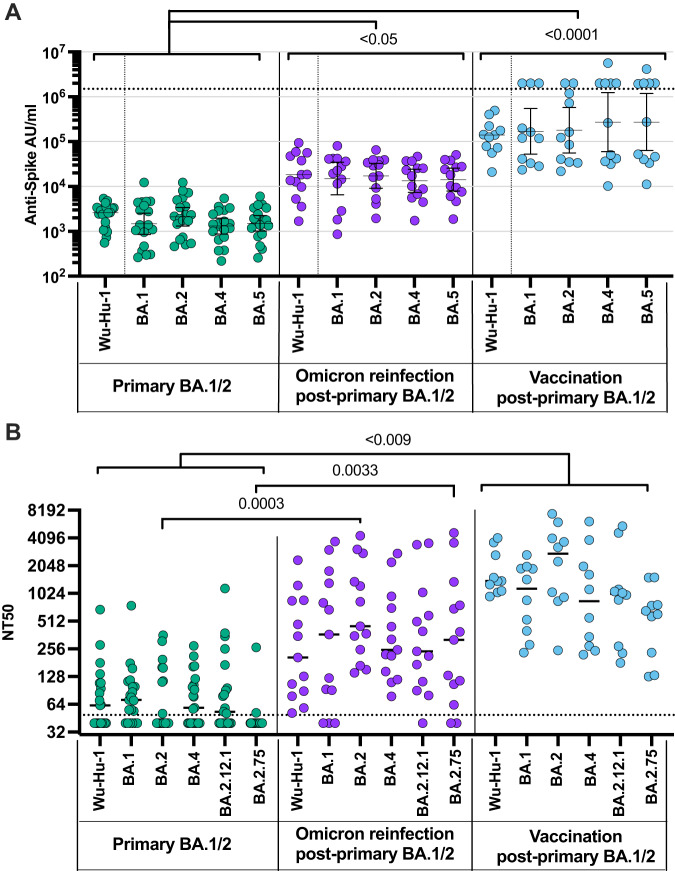
Secondary antigenic challenge enhances antibody levels and neutralisation following primary Omicron infection. **A** Antibody levels specific for spike from Wuhan-Hu-1 and Omicron BA.1, BA.2, BA.4, and BA.5 were determined in children following primary BA.1/2 Omicron infection (green; *n* = 19), Omicron reinfection (purple; *n* = 13) and COVID-19 vaccination (blue; *n* = 11) after primary BA.1/2 infection. **B** Neutralisation of pseudovirus-bearing ancestral B.1 or Omicron variant spike protein in children following primary Omicron BA.1/2 infection (green; *n* = 19), Omicron BA.4/5 reinfection (purple; *n* = 13) or following COVID-19 vaccination after primary BA.1/2 infection (blue; *n* = 11). Data were presented as NT50, the titre at which infectivity had been reduced by 50% relative to controls. Dashed lines indicate the limit of detection. Kruskal–Wallis test with Dunn’s multiple comparisons test, bars indicate geometric mean ±95% CI. Data were presented as arbitrary units (AU)/ml. Source data are provided as a Source Data file.

The magnitude of antibody response and relative neutralisation activity was then determined against ancestral spike and Omicron subvariants following primary Omicron (*n* = 20), Omicron reinfection (*n* = 13) or vaccination after primary infection (*n* = 10). Antibody levels (Fig. 2A) and neutralisation capacity (Fig. 2B) were substantially enhanced against all variants following reinfection or vaccination.

These findings indicate that a second SARS-CoV-2 antigen challenge following primary Omicron infection, either in the form of reinfection or vaccination, markedly boosts spike-specific antibody magnitude and function against current Omicron subvariants.

### Primary vaccination elicits antibodies against Omicron which are not enhanced following breakthrough Omicron infection

15 children within the cohort had received mRNA COVID-19 vaccines prior to the emergence of Omicron. As the vaccine contains the Wuhan-Hu-1 spike we next assessed Omicron-specific antibody titre in this cohort. Additionally, sera were obtained from six children with an Omicron breakthrough infection after vaccination. 10/15 and 4/6 children, respectively, had received two BNT162b2 vaccine doses.

**Fig. 3:**
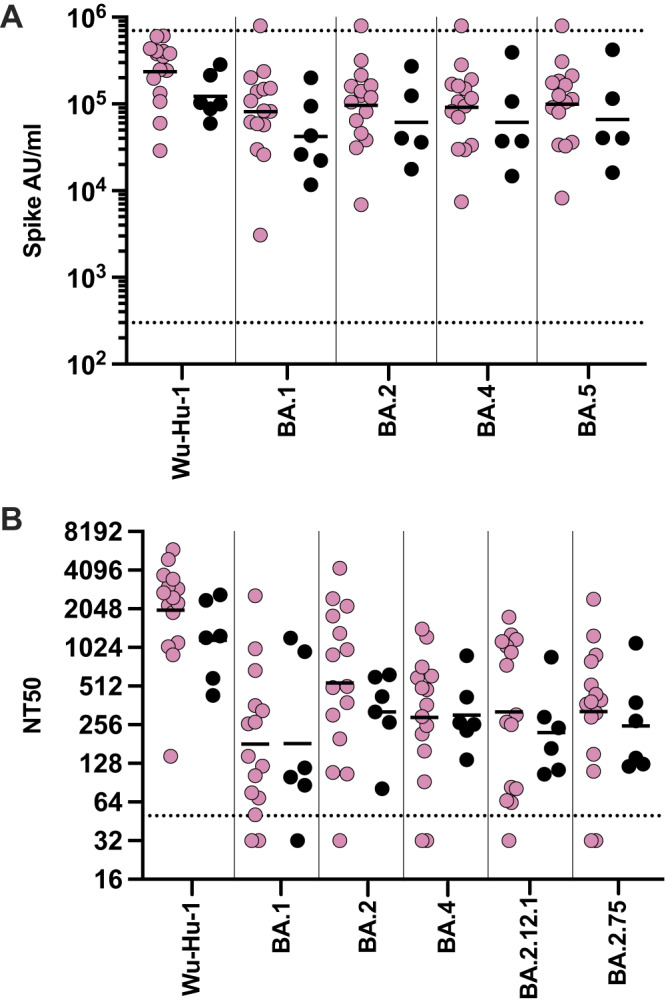
COVID-19 vaccine induces strong antibody responses that are not enhanced following breakthrough infection. **A** Antibody levels specific for spike from Wuhan-Hu-1, and Omicron BA.1, BA.2, BA.4, and BA.5 were determined in children following vaccination (pink, *n* = 15) or BA.1/2 Omicron breakthrough infection (black, *n* = 6). Data were presented as arbitrary units (AU)/ml. **B** Neutralisation of pseudovirus-bearing ancestral B.1 or Omicron variant spike protein in children following vaccination (pink, *n* = 15) or BA.1/2 Omicron breakthrough infection (black, *n* = 6). Dashed lines indicate the limit of detection, bars indicate the geometric mean. Data were presented as NT50, the titre at which infectivity had been reduced by 50% relative to controls. Source data are provided as a Source Data file.

Vaccines were strongly immunogenic and Omicron-specific antibody responses following one or two vaccine doses were 21-fold and 40-fold higher than seen after primary natural infection. Of note, prior infection history was not known and it is possible that some children had been naturally infected prior to vaccination. Antibody responses following Omicron breakthrough infection after vaccination were next assessed and seen to be comparable to those seen in the total vaccine group with no evidence of boosting from a recent infection, potentially due to a ceiling effect on antibody titre (Fig. 3A and Supplementary Fig. 4D, E). Matched pre-infection samples were not available from children with breakthrough infection.

Neutralisation titres were also similar between the two groups and comparable to those seen following dual-Omicron infection (Fig. 3B).

These data show that primary series vaccination in children induces an Omicron-specific antibody response that is higher than seen after Omicron secondary infection whilst Omicron breakthrough infection does not appear to further enhance humoral immunity.

### Initial Omicron or pre-Omicron variant challenge determines the long-term balance of antibody binding

We next determined how an initial infection with Omicron or a pre-Omicron virus acted to determine the relative balance of antibody binding against these two major viral lineages and to what extent this was modulated by a subsequent challenge with Omicron reinfection or vaccination with the Wuhan-Hu-1 spike protein.

**Fig. 4:**
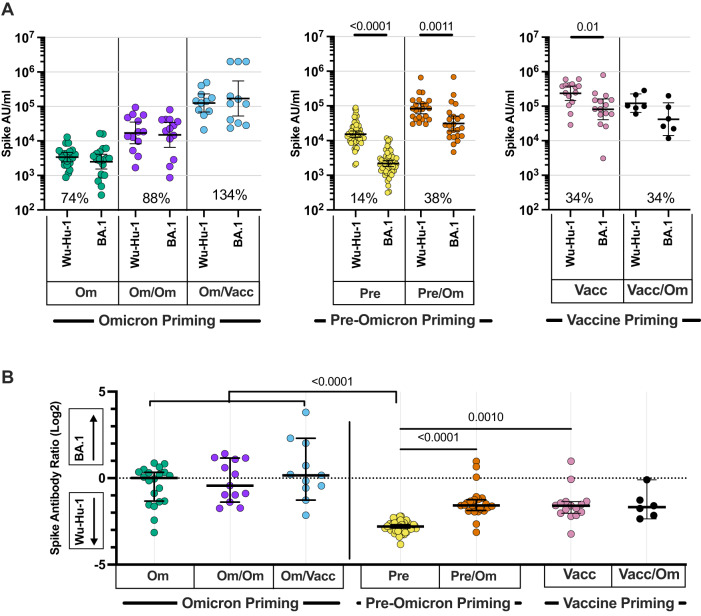
Relative antibody binding to Omicron or ancestral spike protein in relation to primary virus or vaccine challenge. **A** Antibody binding against ancestral Wuhan-Hu-1 or Omicron BA.1 spike in children following three different forms of initial primary antigenic challenge. (i) Initial primary Omicron infection (green; *n* = 19) and subsequent Omicron reinfection (purple; *n* = 13) or vaccination (blue; *n* = 11); (ii) initial primary pre-Omicron infection (yellow; *n* = 54), followed by secondary Omicron BA.1/2 infection (orange; *n* = 23); iii) primary vaccination (pink; *n* = 15) followed by breakthrough Omicron infection (black; *n* = 6). Inset percentages indicate the geometric mean BA.1 antibody level in respect to the Wuhan-Hu-1-specific antibody level. Data were presented as arbitrary units (AU)/ml. Two-tailed paired Wilcoxon test, bars indicate geometric mean ±95% CI. **B** Ratio of antibody binding against Omicron or ancestral Wuhan-Hu-1 spike (Omicron/ancestral) in children following initial primary Omicron or pre-Omicron infection, or vaccination as (**A**). Kruskal–Wallis test with Dunn’s multiple comparisons test, bars indicate median ±95% CI. Source data are provided as a Source Data file.

The median relative ratio of antibody binding against Omicron or pre-Omicron Wuhan-Hu-1 spike was 1.0 following primary Omicron infection indicating a broadly equivalent response. This value increased slightly to 0.7 following Omicron reinfection. Of note, the ratio increased to 1.1 following vaccination showing that vaccine challenge with pre-Omicron spike could not deviate antibody responses away from Omicron recognition (Fig. 4A, B).

A very different pattern was observed in children who had an initial infection with pre-Omicron virus. Here the Omicron:pre-Omicron binding ratio was only 0.14 following primary infection, indicating a strong deviation of recognition towards the ancestral spike. This increased only modestly to 0.34 following Omicron infection. A similar profile was seen in children following primary vaccination or Omicron breakthrough infection with values of 0.34 or 0.31 respectively  (Fig. 4A, B).

These data demonstrate that immune imprinting by the primary viral or vaccine exposure determines the profile of the antibody response following subsequent SARS-CoV-2 challenge.

### SARS-CoV-2-specific cellular responses are similar following differential primary SARS-CoV-2 exposure

**Fig. 5:**
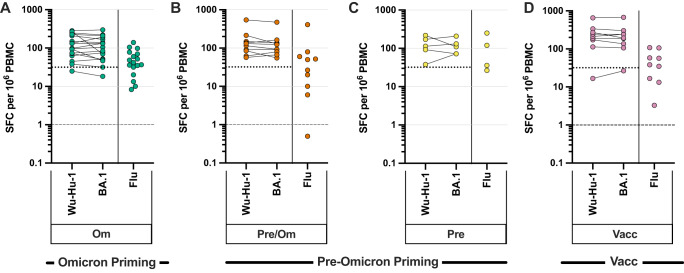
SARS-CoV-2-specific T cell responses are induced reliably following primary Omicron infection and not boosted further by reinfection or vaccination. IFNγ ELISpot was used to assess cellular response to ancestral and Omicron sequence spike peptide pools. Peptides from Influenza (Flu) were included as a control. Children with primary Omicron infection (**A**, green, *n* = 17), Omicron secondary infection (**B**, orange, *n* = 10), pre-Omicron infection sampled prior to the Omicron wave (**C**, yellow, *n* = 5), or vaccinated children (**D**, pink, *n* = 9). Negative control samples including pre-pandemic paediatric blood samples and DMSO alone incubation were used to define the background level of 32 sfc/10^6^ PBMC (16), dotted line. The dashed line indicates the limit of detection. Results are presented as spot-forming cells (sfc) per 10^6^ PBMC. Source data are provided as a Source Data file.

T-cell responses are critical in the control of SARS-CoV-2 but have not yet been assessed in children following Omicron infection. Importantly, spike-specific cellular responses were present in 94% (16/17) of children following primary Omicron infection (Fig. 5A). T-cell responses were also observed in all children following secondary Omicron infection and the magnitude was broadly equivalent (Fig. 5B). Furthermore, cellular immunity was comparable against peptide pools from either the ancestral Wuhan-Hu-1 or Omicron BA.1 spike protein. Omicron-specific cellular immunity was also assessed in historical samples from 5 children who had been infected with pre-Omicron variant, and again, no difference was seen in the magnitude of ancestral or Omicron-specific response (Fig. 5C). As such, T cell immunity against Omicron is comparable following infection with either Omicron or pre-Omicron virus. Analysis of vaccinated children showed cellular responses in 92% of donors (17/19) and again the magnitude was comparable to that seen following natural infection (Fig. 5D).

These data show that a SARS-CoV-2-specific cellular response develops robustly following primary Omicron infection and is broadly cross-reactive against Omicron and pre-Omicron spike protein. In marked contrast to the humoral response, the magnitude of cellular immunity was not significantly boosted by secondary infection or vaccination.

## Discussion

Omicron SARS-CoV-2 is now an endemic infection and it is important to understand immune responses following infection in children. Our findings highlight a range of unique features regarding Omicron-specific immune responses that have potential implications for the trajectory of the pandemic and future vaccine strategies in this age group.

We initially characterised the immune response following primary Omicron infection where a striking feature was the low magnitude and functional quality of the antibody response. Indeed, a quarter of children failed to demonstrate neutralisation activity against BA.1 despite recent infection, and this concurs with other recent findings^11^. These data explain poor protection against reinfection and it was noteworthy that 54% showed serological evidence of reinfection within 3 months, a time period which encompassed the emergence of the BA.4/5 subvariants^6^. A pattern of frequent reinfection is also seen with the four endemic alpha- and beta-HCoV which circulate widely and generally elicit mild upper respiratory tract infection in children^21,22^. Omicron also preferentially replicates within the upper airways and this may underpin a somewhat less severe clinical profile^23–26^ and relatively muted humoral response^27^.

Although primary HCoV infections elicit modest antibody responses that are unable to prevent recurrent infection^20,22,28,29^, repeated infections progressively drive maturation of the antibody response which typically achieves adult levels towards the onset of puberty^20,28,29^. As such we were interested to assess the immunological impact of Omicron reinfection and found that this enhanced antibody titres and neutralisation activity by sixfold. We were also able to assess the impact of the second antigen challenge through vaccination and the observed marked improvement in humoral immunity against all variants is encouraging for potential future protection.

Primary infection with a pre-Omicron virus elicited much stronger humoral immunity against the ancestral virus but notably antibody responses against Omicron spike were comparable to those seen in children after primary Omicron infection. Secondary infection with Omicron in these children delivered a particularly strong antibody boost with impressive neutralisation and exceeded values observed with dual-Omicron infection. Pre-Omicron viruses are thus strongly immunogenic for humoral immunity in children and it is interesting to speculate that this may contribute to the development of multisystem inflammatory syndrome (MIS-C) which can be associated with auto-antibody production^30^.

**Fig. 6:**
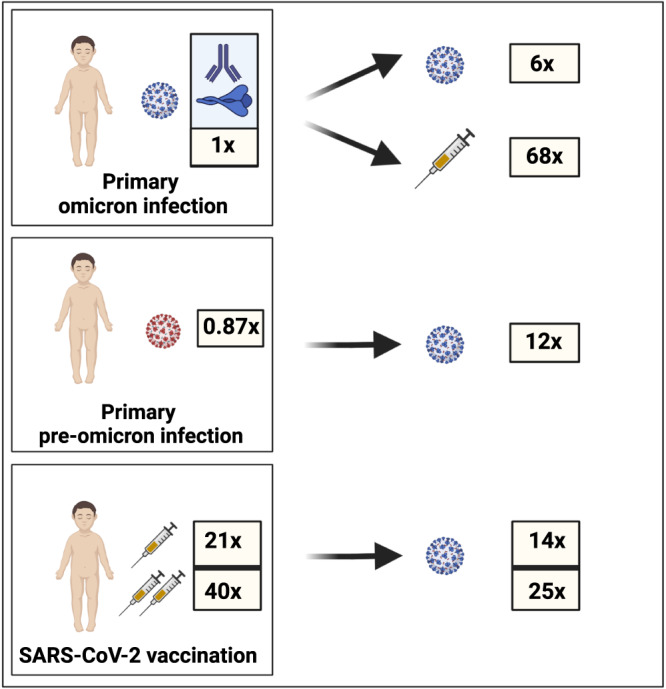
Relative magnitude of omicron-specific antibody response following single or dual SARS-CoV-2 infection or vaccination. Values are given in relation to the magnitude following primary Omicron infection. Values for vaccination show response after one or two vaccine doses.

COVID-19 vaccines are now deployed widely in children and initial vaccination also elicited particularly strong antibody responses. Indeed, a single vaccine dose elicited values that were higher than seen following natural dual infection and this value was doubled following a second dose (Fig. 6), revealing potent vaccine immunogenicity in children despite incomplete clinical protection against infection^6,31^.

Immune imprinting can limit the capacity to respond to variant antigen challenge^32–34^ and is of considerable concern for long-term protection against SARS-CoV-2 variants. Our prospective assessment of antibody specificity allowed a unique opportunity to address this question and we find that immune imprinting has a profound effect on long-term humoral immunity in children. In particular, those who initially experience Omicron infection have a comparable relative antibody response against Omicron and ancestral variants following primary infection and this pattern is maintained following subsequent challenges with either Omicron (reinfection) or ancestral (vaccination) spike. This profile differs somewhat from a predominant Omicron-focussed antibody response reported in adults following primary infection^13^. In contrast, those who are initially exposed through pre-Omicron infection or vaccine develop humoral immunity deviated markedly towards the ancestral spike protein and this profile also remains broadly stable following challenges with Omicron secondary infection or breakthrough infection after vaccination (Fig. 6). This profile of ‘antigenic seniority’ is also seen following primary influenza infection and results in lifelong imprinting of immune responses that can impact clinical protection against influenza A subtypes many decades later^35,36^. Our findings show that similar imprinting of antigenic seniority occurs following SARS-CoV-2 infection in children, the future consequence of which is currently unknown.

However, it is reassuring that Omicron-specific antibody and neutralisation titres were increased following Omicron secondary infection or vaccination and this is compatible with recent murine fate mapping experiments which show that de novo B cell responses against Omicron could develop due to the degree of antigenic divergence between the variants and that these primary clones targeted novel epitopes within the Omicron domain. We thus provide evidence suggesting that, whilst antigenic seniority is evident, this may not functionally impinge on future protective responses in children.

Cellular immunity is an important mediator of protection against severe COVID-19^37^. We find that robust T cell responses develop following either Omicron or pre-Omicron infection. Furthermore, and in direct contrast to humoral immunity, the magnitude of these responses is comparable. This response was also stable after reinfection and similar to that seen following vaccination. As such, cellular immunity appears to be highly sensitive to SARS-CoV-2 infection and may reach a relative plateau following a range of differential initial antigen exposures. Cellular responses against Omicron and ancestral virus were broadly comparable, reflecting the ability of cellular immune recognition to tolerate viral mutations that evade humoral recognition^38^. T-cell responses against endemic HCoV are also robust and somewhat higher in younger people^39^, further suggesting a differential age-dependent priming of immunity against coronaviruses.

Limitations of the study include the absence of samples from vaccines prior to infection and the lack of prospective viral screening to accurately define reinfection following the national withdrawal of community and home testing for SARS-CoV-2 after March 2022.

Our results provide important insights into SARS-CoV-2 immune responses in children and suggest that the Omicron variant of SARS-CoV-2 has evolved to represent a fifth endemic coronavirus infection immunologically. These findings have implications for the future course of the COVID-19 pandemic and its impact on children. Whilst initial Omicron exposure does not reliably generate protective immunity against reinfection this is enhanced following re-exposure and is broadly comparable against Omicron subvariants. Robust cellular responses should support clinical protection against severe disease. Furthermore, the nature of the initial SARS-CoV-2 exposure dictates the subsequent profile of antibody response to antigen challenge and may direct the lifelong pattern of response to SARS-CoV-2 variants and vaccines. Immune imprinting will act as an important individual determinant of humoral immunity, the long-term immunological and clinical importance of which is uncertain.

## Methods

### Sample collection

Samples were collected from two prospective studies within the paediatric population.

The SARS-CoV-2 surveillance in primary schools (sKIDs) study of children aged 6–14 years was a cross-sectional 5–10 ml blood collection study initiated in June 2020 by the United Kingdom Health Security Agency (UK-HSA) after schools reopened following the easing of national lockdown (https://www.gov.uk/guidance/covid-19-paediatric-surveillance). Further collections for this study were undertaken after 21 months (March/April 2022) and 24 months (June/July 2022).

The Born in Bradford study is a prospective longitudinal collection of 5–10 ml blood samples from children aged 11–14 years in Bradford, UK undertaken to study SARS-CoV-2 immune responses^19^. Initial sampling was undertaken in November 2020 and three additional samples from donors were collected at 6 monthly intervals until November 2022.

Omicron infection was determined through linkage with the national SARS-CoV-2 testing database (SGSS) held by UK-HSA and the NIMS database, which records all COVID-19 vaccinations in England, was used to obtain records of COVID-19 vaccination and vaccine manufacturer for each dose. These data were accessed in May 2022.

No statistical methods were used to predetermine sample sizes. Researchers were blinded to the status of donors before ELISpot and serological assessment.

Ethical review for the sKIDs study was provided by the PHE Research Ethics and Governance Group (PHE R&D REGG ref. no. NR0209). Ethical Review of The Born in Bradford study was provided by the National Health Service Health Research Authority Yorkshire and the Humber (Bradford Leeds) Research Ethics Committee; REC reference: 16/YH/0320. Children and parents or guardians were provided with age-appropriate information sheets prior to enrolment. Written informed consent was obtained from all parents or guardians of all participants.

### PBMC and plasma preparation

Lithium Heparin blood tubes were processed within 24 h of collection. Briefly, tubes were spun at 300 g for 10 min prior to removal of plasma which was then spun at 800×*g* for 10 min and stored as aliquots at −80 °C. The remaining blood was diluted with RPMI and PBMC isolated on a Sepmate ficol density gradient (Stemcell), cells were washed with RPMI and rested in RPMI + 10% batch-tested FBS for a minimum of 4 h prior to cellular assays.

### Serological analysis of SARS-CoV-2-specific immune response

Quantitative IgG antibody titres were measured using Mesoscale Diagnostics multiplex assays; Coronavirus Panel 22 (K15559U), 24 (K15575U), 25 (K15583U) and 27 (K15606U) following the manufacturer instructions. Briefly, samples were diluted 1:5000 in supplied diluent 100 and added wells of the 96 well plates were alongside reference standards and controls. After incubation, plates were washed five times with the provided wash buffer and anti-IgG-Sulfo tagged detection antibody diluted 1:200 diluent added. Plates were washed, ECL reagent added and were immediately read using a MESO QuickPlex SQ 120 system. Data was generated by Methodological Mind software and analysed with MSD Discovery Workbench (v4.0) software. Data were presented as arbitrary units (AU)/ml determined relative to an eight-point standard curve generated on each MSD plate with standards provided by MSD. Cut-offs were previously defined against pre-pandemic adult and paediatric samples^17^. Wuhan-Hu-1 RBD-specific antibody titres above or below the geometric mean titre was used to define primary or secondary Omicron infection respectively.

### Pseudotype-based neutralisation assays

Constructs and 293-ACE2 cells were previously described in refs. ^17,40^. Neutralising activity in each sample was measured against pseudovirus displaying a SARS-CoV-2 spike, either B.1 sequence or B.1.1.529:BA.1, BA.2, BA.4, BA.2.12.1 or BA.2.75 sequence, by a serial dilution approach. Each sample was serially diluted in triplicate from 1:50 to 1:36450 in complete DMEM prior to incubation with ~1 × 10^6^ CPS (counts per second) per well of HIV (SARS-CoV-2) pseudotypes, incubated for 1 h, and plated onto 239-ACE2 target cells. After 48–72 h, luciferase activity was quantified by the addition of Steadylite Plus chemiluminescence substrate and analysis on a Perkin Elmer EnSight multimode plate reader (Perkin Elmer, Beaconsfield, UK). Antibody titre was then estimated by interpolating the point at which infectivity had been reduced to 50% of the value for the no serum control samples.

### IFNγ ELISpot

T cell responses were measured using an IFNγ ELISpot Pro kit (3420-2APT, Mabtech) as previously described in ref. ^17^. Pepmixes pool containing 15-mer peptides overlapping by 10aa from either SARS-CoV-2 spike S1 or S2 domains from the Wuhan-Hu-1 (PM-WCPV-S, protein ID: P0DTC2, https://www.uniprot.org/uniprotkb/P0DTC2/) or Omicron (PM-SARS2-SMUT08, B1.1.529; BA.1) variant were purchased from JPT technologies. Overlapping pepmixes from influenza A matrix protein 1, California/08/2009 (H1N1) (PM-INFA-MP1-H1N1, protein ID: C3W5Z8, https://www.uniprot.org/uniprotkb/C3W5Z8) and A/Aichi/2/1968 H3N2 (PM-INFA_MP1, protein ID: Q67157, https://www.uniprot.org/uniprotkb/Q67157) were purchased from JPT Peptide Technologies and combined as a relevant control. All peptide were dissolved in Dimethyl sulfoxide (Sigma), aliquoted and stored at −20 °C.

Briefly, fresh PBMC were rested overnight prior to assay, prior to cell addition ELISpot plates were washed with 0.22 μm filtered PBS, and blocked for 1 h with RPMI + 10%FBS, media was removed and replaced with 50 μl volume of media to which 10 μl of peptide or controls were added prior to 0.25–0.3 × 10^6^ PBMC in duplicate per well containing either pep-mix (at a final concentration of 1 μg/ml per peptide), or DMSO (negative) control, or a single well-containing anti-CD3 (positive). Samples were incubated for 16–18 h in a humidified incubator at 37 °C, 5% CO_2_. Plates were then washed with PBS and incubated with the supplied anti-IFNγ-ALP conjugated secondary reagent, after further washing plates were developed with the supplied BCIP/NBT-plus substrate, following the manufacturer’s instructions and read using an AID plate reader (AID), with AID ELISpot software version 7.

### Data visualisation and statistics

Data were initially exported into Microsoft Excel 2019 for Mac. Data were visualised and statistical tests, including normality tests, performed as indicated using GraphPad Prism v9 software. Only results found to be significant (*p* < 0.05) are displayed.

### Reporting summary

Further information on research design is available in the Nature Portfolio Reporting Summary linked to this article.

## Supplementary information

Supplementary Information

Peer Review File

Reporting Summary

## Data Availability

All data generated or analysed during this study are included in this published article and its supplementary information files or from the corresponding author upon request. Source data are provided with this paper. The NIMS database is not publicly available due to the sensitive nature of the information contained, however, information relating to vaccination status is provided in the manuscript. Source data are provided with this paper.

## Source data

Source Data
