# Supplementary material for: Immunological imprinting of humoral immunity to SARS-CoV-2 in children
Source: Nat Commun. 2023 Jun 29;14:3845. doi: 10.1038/s41467-023-39575-2 (PMC10310754; doi:10.1038/s41467-023-39575-2)
Supplement: Supplementary file 1 — Supplementary Information [file 41467_2023_39575_MOESM1_ESM.pdf]

Immunological imprinting of humoral immunity to SARS-CoV-2 in children

Supplementary Tables and Figures

	Omicron Infection	Vaccinated	Omicron infection after Vaccination	Vaccination after primary Omicron
**Number**	43	15	6	5
**Mean Age (range)**	10.7 (6-14)	12 (7-14)	11.4 (6-13)	10 (9-11)
**Sex**	22 female<br>19 male<br>1 n/a	10 female<br>4 male	2 female<br>4 male	2 female<br>3 male
**Days after infection (range)**	78 (10-173)	-	47 (9-95)	141 (100-158)
**Days post vaccine**	-	80 (43-178)	136 (86-222)	46 (12-81)

Supplementary Table 1. Cohort Demographics

Cohort		Antibody (arbitrary units /ml)			
		Spike-Wuhan -Hu-1	Spike-Omicron BA.1	RBD- Wuhan -Hu-1	RBD- Omicron BA.1
**Pre-Omicron**	**N=54**	15333 (12360-19,020)	2188 (1770-2710	4546 (3580-5,770)	538 (426-680)
**Primary BA.1/2 infection**	**N=20**	3401 (2470-4680)	2504 (1530-4110)	470 (290-770)	411 (245-640)
**Secondary BA.1/2 infection**	**N=23**	82850 (59254-115852)	33187 (18530-59440)	31922 (21510-47370)	7774 (4080-14820)
**Vaccinated**	**N=15**	235899 (145573-382273)	81320 (40874-161788)	n.d.	n.d.
Vaccinated – First dose only	*N=5*	*207307 (49961-860196)*	*53710 (7000-412135)*	n.d.	n.d.
Vaccinated – Second dose only	*N=10*	*251642 (145643-434788)*	*100061 (47911-208978)*	n.d.	n.d.
**Omicron post vaccination**	**N=6**	122202 (65947-226445)	42091 (14195-124808)	n.d.	n.d.
**Vaccination post-primary BA.1/2**	**N=11**	126676 (69341-231419)	169722 (52685-546751)	n.d.	n.d.
**Secondary exposure post-primary BA.1/2**	**N=13**	17096 (8289-32257)	15001 (6523-34498)	n.d.	n.d.

Supplementary Table 2. Antibody levels in cohorts. Geometric mean (95% CI of geo. mean).

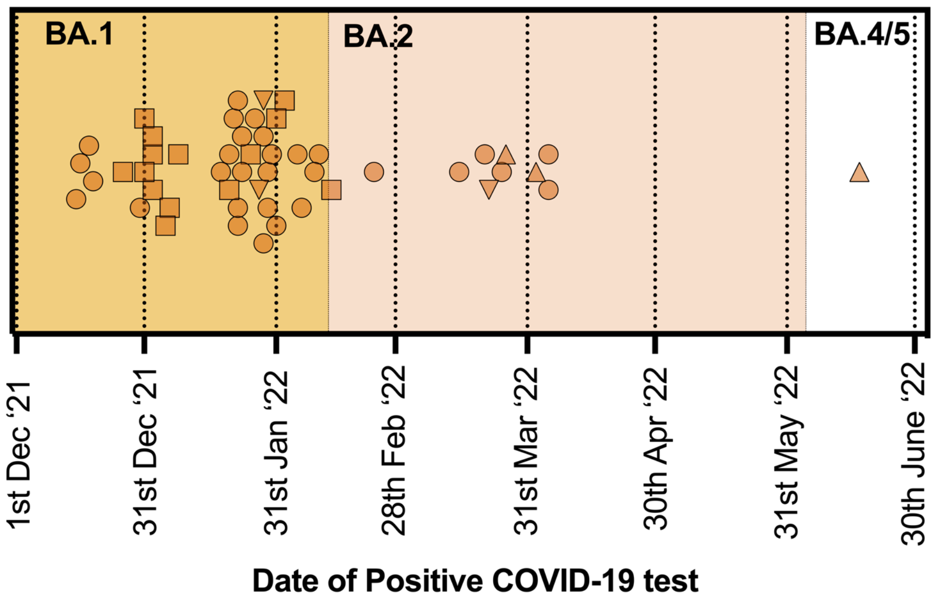

Supplementary Figure 1. Timing of SARS-CoV-2 Infection in relation to Omicron variant prevalence.

Timing of positive COVID-19 test results for sKIDs (circles) and Born in Bradford (squares) samples. Triangles indicate breakthrough infections after vaccination. The majority of infections occurred during the BA.1 wave (yellow) whilst the pink shaded area indicates the period in which BA.2 comprised over 50% of sequenced infections nationally. The emergence of BA.4/5 was seen from June 2022 onwards.

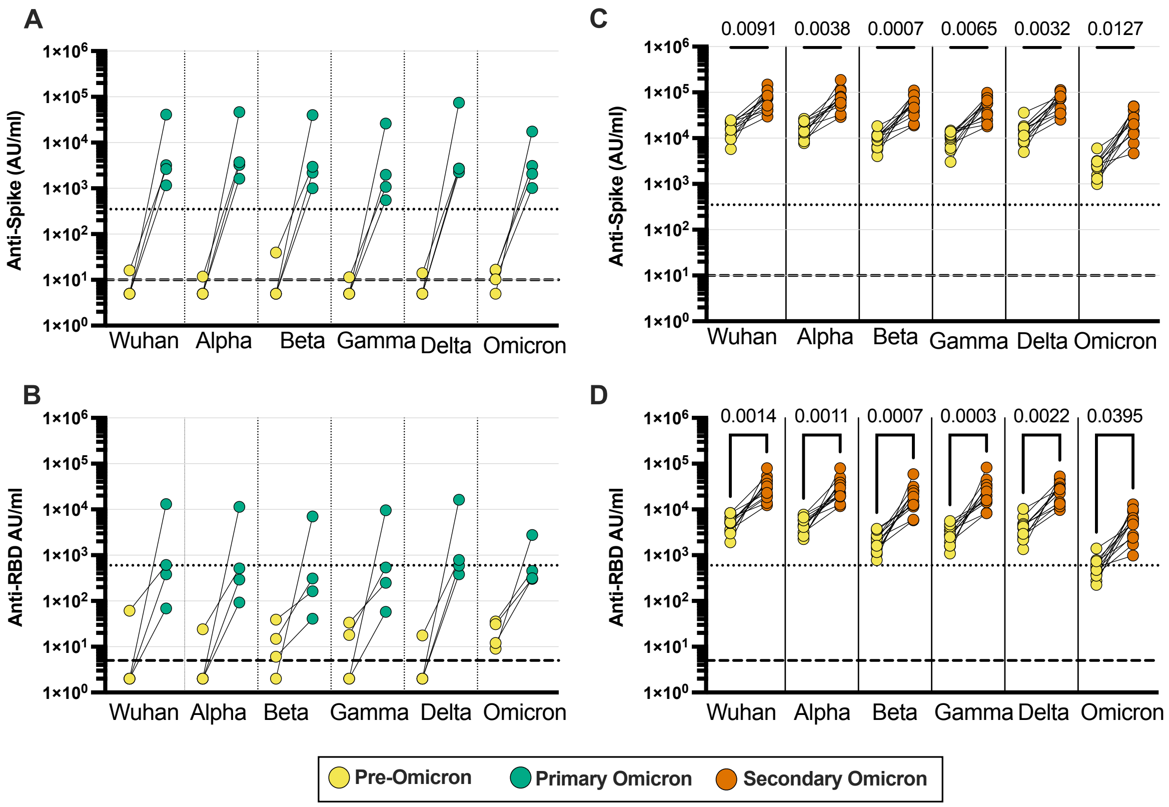

Supplementary Figure 2. Profile of SARS-CoV-2-specific antibody response following primary or secondary Omicron infection

SARS-CoV-2-specific antibody responses were determined in longitudinal plasma samples from children with BA.1/2 infection. Spike (A) and RBD-specific (B) antibody binding against viral variants in samples from children (n=4) who were seronegative prior to Omicron infection (yellow) and following BA.1/2 infection (green).

Spike (C) and RBD-specific (D) antibody binding against viral variants in samples from children (n=11) who were seropositive prior to BA.1/2 infection (yellow) and following BA.1/2 infection (orange).

Data are presented as arbitrary units (AU)/ml. Dotted lines indicate sero-positive cut-offs as determined for Wuhan-Hu-1-specific antibody response; dashed lines indicate below limit of detection. One-way Friedman Test with Dunn's multiple comparisons test. Source data are provided as a Source Data file.

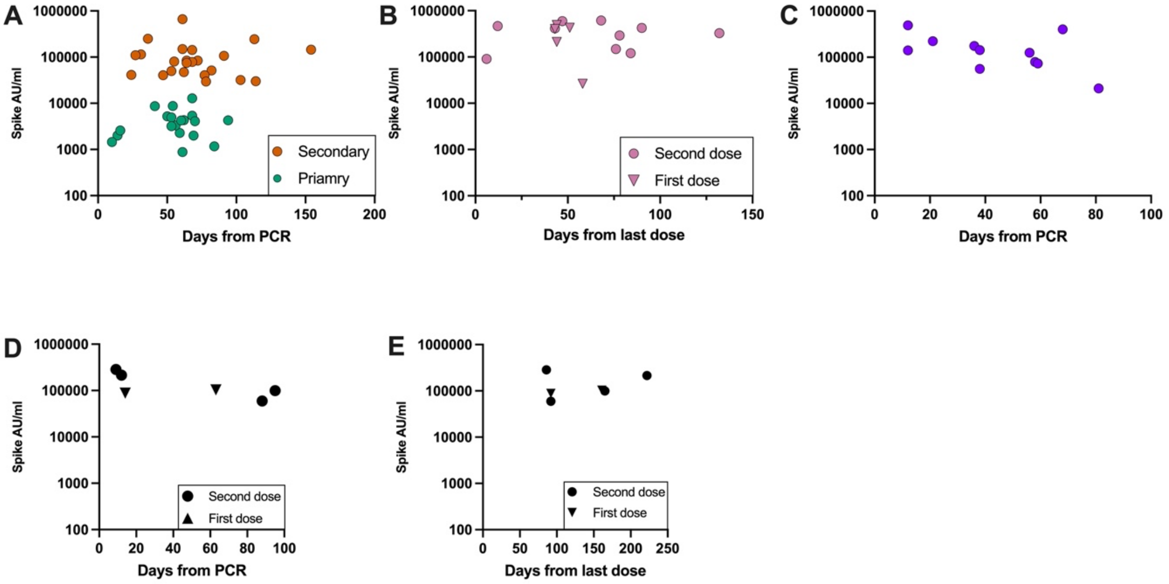

Supplementary Figure 3. Antibody titre in respect of time from infection or vaccination.
The prospective nature of sample collection was such that children were studied at varying timepoints after infection or vaccination. Antibody titre against ancestral B.1 spike was therefore plotted against time since antigen challenge to assess the potential impact of antibody waning.

A) Time from primary (green) or secondary (orange) Omicron infection.
B) Time from a first (triangle) or second (circle) COVID-19 vaccine dose.
C) Time from single vaccine dose in children with prior Omicron infection.
D) Time from Breakthrough infection or (E) last vaccine dose in vaccinated children who experienced a breakthrough Omicron infection.
Data are presented as arbitrary units (AU)/ml. Source data are provided as a Source Data file.

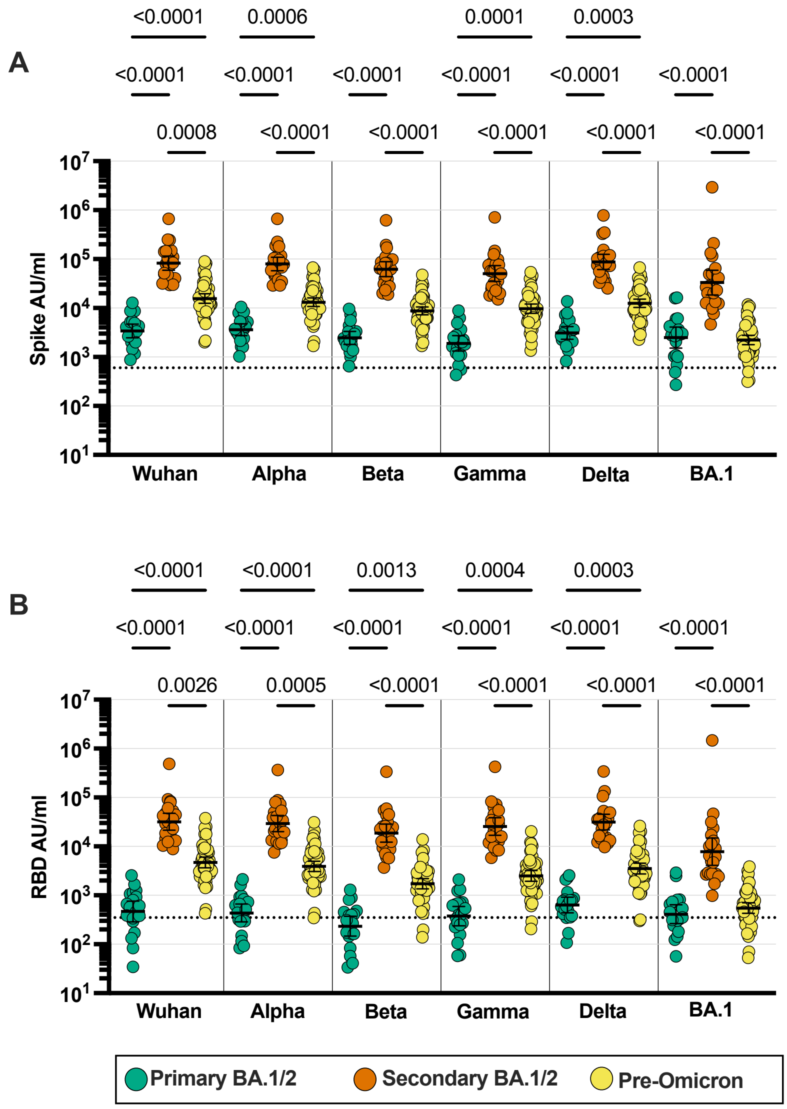

Supplementary Figure 4. Relative antibody titre against SARS-CoV-2 viral variants following primary or secondary Omicron infection.

Samples from children (n=43, aged 6-14 years) recently infected with Omicron BA.1/2 variant were assessed on the MSD-platform for antibodies against Spike protein (A) and RBD-domain (B) from SARS-CoV-2 variants as indicated. Donors were divided in those with primary SARS-CoV-2 infection (green, n=20) or secondary SARS-CoV-2 infection (Orange, n=23). Values are also compared to antibody levels from historical children's samples taken after SARS-CoV-2 infection prior to emergence of Omicron (yellow, n=54, aged 5-14 years). One-way Kruskal-Wallis test with Dunn's multiple comparisons test. bars indicate geometric mean ±95% CI. Data are presented as arbitrary units (AU)/ml. Source data are provided as a Source Data file.

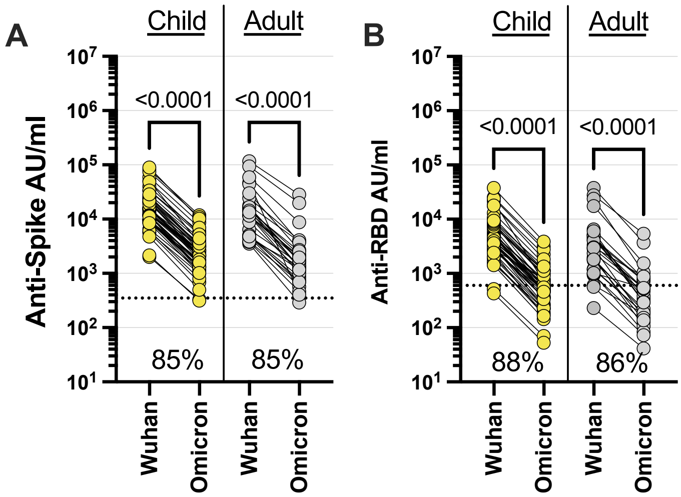

Supplementary Figure 5. Marked reduction in Omicron-specific antibody binding in sera from children with pre-Omicron natural infection

Antibody binding to Wuhan-Hu-1 or BA.1 spike (A) or RBD-domain (B) in seropositive children (n=54, aged 5-14 years) and adults (n=30; aged >18 years), infected and sampled prior to the emergence of Omicron. Lines join individual donors. Inset percentage indicates the average reduction in binding (arb. units (AU)/ml) to Omicron protein compared to Wuhan. One-way Kruskal-Wallis test with Dunn's multiple comparisons test. Dotted lines indicate seropositive cut-offs as determined for Wuhan-specific response. Data are presented as arbitrary units (AU)/ml. Source data are provided as a Source Data file.
