# Supplementary material for: Immunological imprinting of humoral immunity to SARS-CoV-2 in children
Source: Nat Commun. 2023 Jun 29;14:3845. doi: 10.1038/s41467-023-39575-2 (PMC10310754; doi:10.1038/s41467-023-39575-2)
Supplement: Supplementary file 3 — Reporting Summary [file 41467_2023_39575_MOESM3_ESM.pdf]

nature portfolio

Corresponding author(s): Paul Moss

Last updated by author(s): 22/5/22

Reporting Summary

Nature Portfolio wishes to improve the reproducibility of the work that we publish. This form provides structure for consistency and transparency in reporting. For further information on Nature Portfolio policies, see our Editorial Policies and the Editorial Policy Checklist.

Statistics

For all statistical analyses, confirm that the following items are present in the figure legend, table legend, main text, or Methods section.

n/a	Confirmed	
☐	☒	The exact sample size (n) for each experimental group/condition, given as a discrete number and unit of measurement
☐	☒	A statement on whether measurements were taken from distinct samples or whether the same sample was measured repeatedly
☐	☒	The statistical test(s) used AND whether they are one- or two-sided<br>*Only common tests should be described solely by name; describe more complex techniques in the Methods section.*
☐	☒	A description of all covariates tested
☐	☒	A description of any assumptions or corrections, such as tests of normality and adjustment for multiple comparisons
☐	☒	A full description of the statistical parameters including central tendency (e.g. means) or other basic estimates (e.g. regression coefficient) AND variation (e.g. standard deviation) or associated estimates of uncertainty (e.g. confidence intervals)
☐	☒	For null hypothesis testing, the test statistic (e.g. F, t, r) with confidence intervals, effect sizes, degrees of freedom and P value noted<br>*Give P values as exact values whenever suitable.*
☒	☐	For Bayesian analysis, information on the choice of priors and Markov chain Monte Carlo settings
☒	☐	For hierarchical and complex designs, identification of the appropriate level for tests and full reporting of outcomes
☒	☐	Estimates of effect sizes (e.g. Cohen's d, Pearson's r), indicating how they were calculated

Our web collection on statistics for biologists contains articles on many of the points above.

Software and code

Policy information about availability of computer code

Data collection	AID ELISpot Software Version 7, Meso Scale Diagnostics Discovery Workbench version 4.0
Data analysis	Graphpad Prism Version 9, Meso Scale Diagnostics Workbench analysis software version 4.0, Microsoft Excel 2019 for Mac

For manuscripts utilizing custom algorithms or software that are central to the research but not yet described in published literature, software must be made available to editors and reviewers. We strongly encourage code deposition in a community repository (e.g. GitHub). See the Nature Portfolio guidelines for submitting code & software for further information.

Data

Policy information about availability of data

All manuscripts must include a data availability statement. This statement should provide the following information, where applicable:

- Accession codes, unique identifiers, or web links for publicly available datasets
- A description of any restrictions on data availability
- For clinical datasets or third party data, please ensure that the statement adheres to our policy

All data generated or analyzed during this study are included in this published article (and its supplementary information files).
Data from the NIMS database, linked to the NHS database is not publicly available due to the sensitive nature of the information contained, however information relating to vaccination status is provided in the manuscript.

Human research participants

Policy information about studies involving human research participants and Sex and Gender in Research.

Reporting on sex and gender	Details of the reported sex of the participants is provided in supplementary table 1. Sex of participants was self reported, participants were free to respond as not applicable. Given the relatively small sample numbers and relatively uniform immune response in this study and prior studies no formal analysis of sex specific responses in children was undertaken.
Population characteristics	Children were divided based on prior serological evidence of SARS-CoV-2 infection and known SARS-CoV-2 infection with an Omicron variant (mean age 10.7 (range 6-14) years, 22 female, 19 male, 1 n/a). Children were also divided dependent on COVID-19 vaccination status; prior vaccination (mean age 12 (range 7-14) years, 10 female, 4 male), or vaccination following an Omicron primary infection (mean age 10 (range 9-11) years, 2 female, 3 male), as determined from the national immunization database. These groups were subdivided from a single cohort as such are believed to otherwise be closely matched.
Recruitment	Children previously recruited to the Born in Bradford or schools participating in the sKIDs study were asked to participate in the current collection. No selection criteria were used to identify participants, as such no self-selection or other bias in recruitment is believed to have occurred. Children and parents/guardians received age appropriate patient information sheets prior to donation. Informed consent was obtained from children and written informed consent was obtained from parents or legal guardians. Participants did not receive compensation or reward for participation.
Ethics oversight	PHE Research Ethics and Governance Group and National Health Service Health Research Authority Yorkshire and the Humber (Bradford Leeds) Research Ethics Committee.

Note that full information on the approval of the study protocol must also be provided in the manuscript.

Field-specific reporting

Please select the one below that is the best fit for your research. If you are not sure, read the appropriate sections before making your selection.

[x] Life sciences   [ ] Behavioural & social sciences   [ ] Ecological, evolutionary & environmental sciences

For a reference copy of the document with all sections, see nature.com/documents/nr-reporting-summary-flat.pdf

Life sciences study design

All studies must disclose on these points even when the disclosure is negative.

Sample size	Samples were collected prospectively and infection and vaccination history subsequently determined, no predefined methods were used to determine sample size. A total of 150 children were recruited to the sKIDS-22 study. The Born in Bradford study initially recruited 161 children, which were followed longitudinally. This was believed to give a large enough sample size to provide a broad cross sectional view of infection and vaccination histories given the known vaccine uptake and infection rates. Further donors were not available.
Data exclusions	Donors within 7 days of primary vaccination or known infection were excluded due to insufficient time for an immune response to be generated. Results failing quality controls, e.g. high DMSO background in ELISpot, were excluded.
Replication	Where possible assays were performed in duplicate, assays showed good correlation between duplicates. Due to the small volumes of blood from paediatrics donors cellular assays were not repeated.
Randomization	Donors were recruited at random, as such randomization was not relevant.
Blinding	Researchers were blinded to the vaccination and infection histories of sKIDs study participants until initial serological and cellular assays were completed.

Reporting for specific materials, systems and methods

We require information from authors about some types of materials, experimental systems and methods used in many studies. Here, indicate whether each material, system or method listed is relevant to your study. If you are not sure if a list item applies to your research, read the appropriate section before selecting a response.

Materials & experimental systems

n/a	Involved in the study
	☒ Antibodies
	☒ Eukaryotic cell lines
☒	Palaeontology and archaeology
☒	Animals and other organisms
☒	Clinical data
☒	Dual use research of concern

Methods

n/a	Involved in the study
☒	ChIP-seq
☒	Flow cytometry
☒	MRI-based neuroimaging

Antibodies

Antibodies used	Monoclonal SULFO-TAG-conjugated Anti-Human IgG antibody
Validation	Antibody supplied and pre-titrated by Mesoscale diagnostics as part of the COVID-19 serology kits, this antibody has been validated by the manufacture and does not cross react with other immunoglobulin isotypes (IgA, IgE, or IgM)

Eukaryotic cell lines

Policy information about cell lines and Sex and Gender in Research

Cell line source(s)	Human embryonic kidney (HEK) 293 cells were obtained by Brian Willett from Yasu Takeuchi, University College London approximately 2000. HEK293, HEK293T and 293-ACE2 cells were maintained in DMEM supplemented with 10% FCS, 200mM of L-glutamine, 100µgml–1 streptomycin and 100IUml–1 penicillin. HEK293T cells were used for pseudovirus production, cells were transfected with the appropriate SARS-CoV-2 S gene expression vector in conjunction with lentiviral vectors p8.91 and pCSFLW using polyethylenimine (Polysciences). 293-ACE2 target cells were generated by stable transduction of HEK293 cells with pSCRPSY-human ACE2 (hACE2). Selected 293-ACE2 cells were maintained in complete DMEM supplemented with 2-µg/mL puromycin as previously described (https://doi.org/10.1093/infdis/jiaa788).
Authentication	Cells were not authenticated
Mycoplasma contamination	Cell lines were screened for mycoplasma and were negative.
Commonly misidentified lines (See ICLAC register)	NA
